# Characterization of the complete chloroplast genome of *Gymnocladus chinensis* Baill

**DOI:** 10.1080/23802359.2021.1931510

**Published:** 2021-05-27

**Authors:** Xiao Feng, Zhao Yang, Wang Xiu-rong, Ya-yan Zhu

**Affiliations:** a College of forestry, Guizhou University, Guiyang, China; b Institute for Forest Resources & Environment of Guizhou, Guizhou University, Guiyang, China; c Key Laboratory of Forest Cultivation in Plateau Mountain of Guizhou Province, Guizhou University, Guiyang, China; d Key Laboratory of Plant Resource Conservation and Germplasm Innovation in Mountainous Region (Ministry of Education), Guizhou University, Guiyang, China; e Guizhou Academy of Forestry, Guiyang, Guizhou, China

**Keywords:** *Gymnocladus chinensis*, chloroplast genome, phylogenetic analysis

## Abstract

*Gymnocladus chinensis* Baill (Fam.: Leguminosae; Trib.: Caesalpinieae) are widely distributed in China. In this study, we assembled the complete chloroplast (cp) genome of *G. chinensis*. The total cp genome size was 165,315 bp in length, containing a large single-copy region of 92,356 bp, a small single-copy region of 20,449bp, and a pair of inverted repeat regions of 26,255 bp. The all GC content of *G. chinensis* cp was 34.95%. It encodes a total of 105 unique genes, including 75 protein-coding genes, 26 tRNA genes, and four rRNA genes. Seventeen genes contain a single intron, and two genes (*ycf3* and *clpP*) have two introns. Phylogenetic analysis results strongly supported that *G. chinensis* was closely related to *Angylocalyx braunii.*

*Gymnocladus chinensis* Baill (Fam.: Leguminosae; Trib.: Caesalpinieae) is a deciduous tree, thornless, no thorns, hybrid flowers, leaves pinnae opposite, subopposite, or alternate, 5–10 pairs. Its seed oil can be used as industrial oil such as paint. *Gymnocladus chinensis* is an excellent tree species for ecological environment forests, economic forests, urban landscape forests, and rural ‘four sides’ greening (Yuan et al. 2011; Wu et al. 1994–2013).

The chloroplast (cp) genome has a conserved DNA of closed-loop structure, and multiple copies exist in the cell. The seeds of *G. chinensis* were collected in Duyun City, Guizhou Province, China (E:107.879199; N:26.247993), and were germinated and cultivated in the laboratory. The EasyPure^®^ Plant Genomic DNA Kit (TransGen Biotech, Beijing, China) was used to extract total genomic DNA from new leaves. The total DNA was used to generate a DNA library with an average insert size of 400 bp. The library was sequenced using the Illumina NovaSeq platform. The seed specimen was deposited at the Institute for Forest Resources & Environment of Guizhou (http://frerc.gzu.edu.cn/) under the voucher number Gc-001-1. The genome sequence of *G. chinensis* was assembled by GetOrganelle (Jin et al. 2020) and was annotated by CPGAVAS2 (Shi et al. 2019). The annotated cp genome sequence and raw reads have been deposited in Genbank with accession number MW023059.1 and SRR12959829, respectively.

The *G. chinensis* cp genome showed a typical circular double-stranded tetrad structure, which was 165,315 bp, containing a large single-copy (LSC) region of 92,356 bp, a small single-copy (SSC) region of 20,449 bp, two reverse repeated regions (IRa and IRb) of 26,255 bp in length. The GC content of *G. chinensis* cp was 34.95%. It encodes a total of 105 unique genes, including 75 protein-coding genes, 26 tRNA genes, and four rRNA genes. Seventeen genes (*trnK-UUU*, *rps16*, *trnT-CGU*, *atpF*…) contain a single intron, and two genes (*ycf3* and *clpP*) have two introns.

Twenty-one complete cp genome sequences including *G. chinensis* were aligned by MAFFT (Katoh and Standley 2013). Phylogenetic analysis was conducted based on maximum likelihood (ML) analyses implemented in iqtree (Nguyen et al. 2015) under the TVM + F+R3 nucleotide substitution model with 10,000 bootstraps. Phylogenetic relationships analysis suggested that *G. chinensis* is closely clustered with *Angylocalyx braunii* (Fam.: Leguminosae; Trib.: Papilionoideae) (Figure 1). This study provided basic data for the chloroplast genetic resources and population evolution of *G. chinensis*.

**Figure 1.**
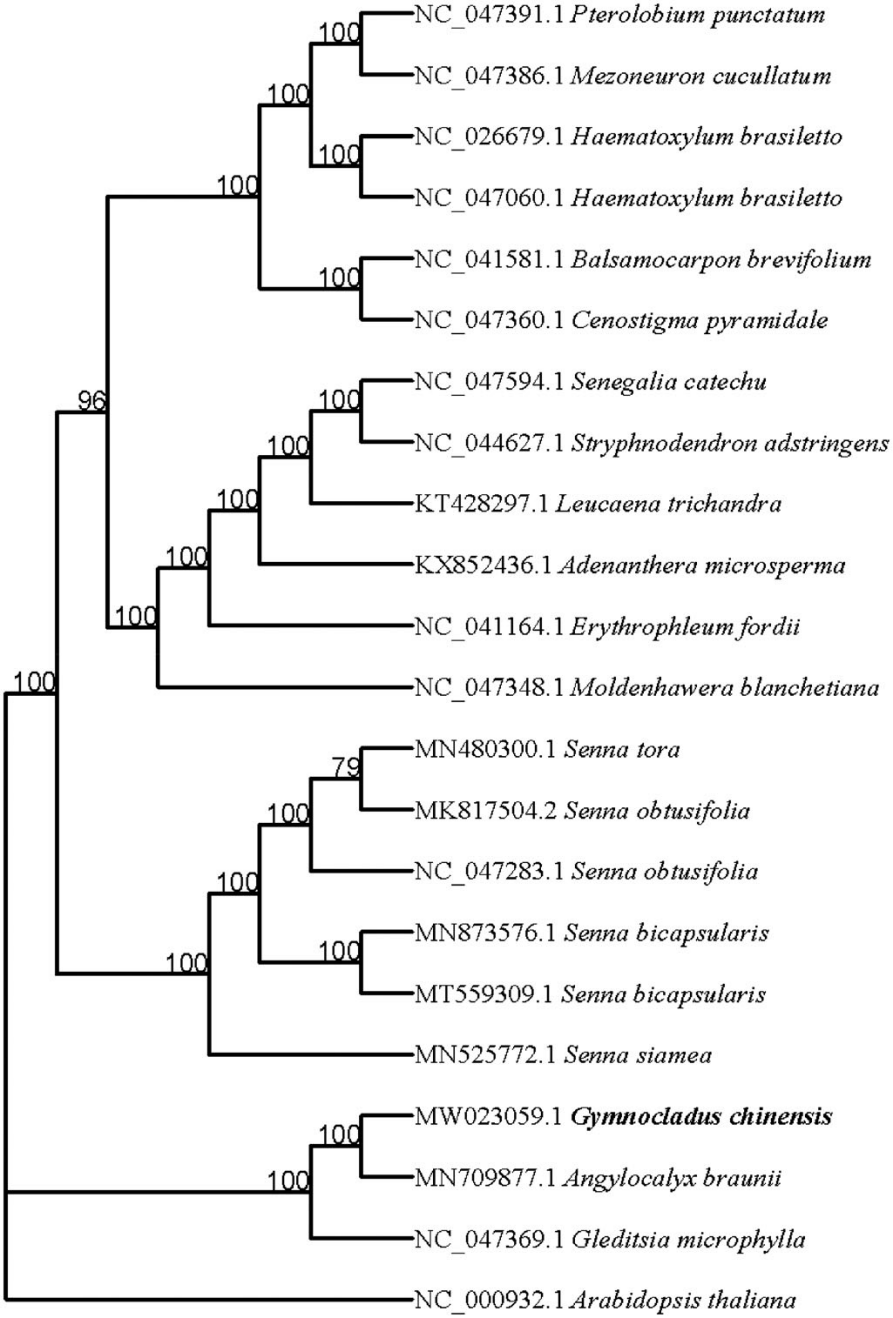
Phylogenetic relationships among 22 complete chloroplast genomes.

## Data Availability

The genome sequence data that support the findings of this study are openly available in GenBank of NCBI (https://www.ncbi.nlm.nih.gov/) under the accession no. MW023059.1. The associated BioProject, SRA, and Bio-Sample numbers are PRJNA672778, SRR12959829, and SAMN16576583 respectively.
